# Whose responsibility? Elder support norms regarding the provision and financing of assistance with daily activities across economically developed countries

**DOI:** 10.1007/s10433-019-00515-z

**Published:** 2019-05-10

**Authors:** Alexander L. Janus, Alison Koslowski

**Affiliations:** School of Social and Political Science, University of Edinburgh, Edinburgh, EH8 9LD UK

**Keywords:** Attitudes, Norms, Elder care, Cross-national research, Social policy

## Abstract

We use 2012 data on economically developed countries from the International Social Survey Program to examine variation in “cultural norms” (at the world region and country levels) and “attitudes” (at the individual level) regarding the appropriate roles of family members and formal providers in both the provision and financing of assistance with daily activities at home. Our analysis has two parts: (1) a descriptive analysis of differences in cultural norms by world region and country (*N *= 25 countries) and (2) a multilevel multinomial logistic regression analysis of the importance of country-level factors in explaining individuals’ elder support attitudes (*N *= 21 countries). In the descriptive analysis, we find substantial variation in cultural norms both between world regions and between countries within all world regions except for the Nordic countries. The multilevel regression analysis points to the importance of two sets of country-level factors—“macrostructural factors” and “cultural–contextual factors”—in explaining individuals’ elder support attitudes. With regard to macrostructural factors, we find, consistent with our hypotheses, greater support for “publicly financed formal assistance” (i.e., the financing of formal assistance is supported by public funds) in countries with higher spending on services. The effects of the cultural–contextual factors are mostly consistent with our hypotheses and suggest the importance of taking into account the wider religious and political context in explaining individuals’ elder support attitudes. We conclude with a discussion of the social scientific and social policy implications of our findings.

**Electronic supplementary material:**

The online version of this article (10.1007/s10433-019-00515-z) contains supplementary material, which is available to authorized users.

## Introduction

While there is a large body of evidence on the macrostructural factors that influence families’ elder support choices (Brandt 2013; Suanet et al. 2012), we know relatively little about cross-national differences in relevant cultural norms. Gerontological research from at least the past 50 years lends support to Tönnies’s (1887/2002) notion that, as *Gemeinschaft* relationships, family relationships reflect normative expectations that shape family members’ affective and behavioral orientations (Ikkink et al. 1999; Silverstein et al. 2006). The available evidence suggests that such normative expectations, including those that are relevant to families’ elder support choices, vary across countries (Daatland and Herlofson 2003; Daatland et al. 2011; Dykstra and Fokkema 2011; Glaser et al. 2004; Yeh et al. 2013).

“Cultural norms” (at the macrolevel) and “attitudes” (at the individual level) regarding elder support are a social policy concern, because they are associated with families’ actual elder support choices (Lin and Yi 2011; Lowenstein and Daatland 2006; Silverstein et al. 2006) and have implications for how different elder support arrangements are experienced (Lee et al. 2002). Cultural norms and individuals’ attitudes about the appropriate roles of family members and formal providers (e.g., government agencies), which we focus on in this study, are especially relevant, because in many economically developed countries formal providers play an important role either in supplementing the efforts of family helpers or in supporting disabled older people without available informal support resources (Litwin and Attias-Donfut 2009; Motel-Klingebiel et al. 2005). Countries have been reassessing their elder support strategies in the face of common demographic and fiscal pressures (Misra et al. 2006; Simonazzi 2009), and information about the elder support norms in a country indicates the direction of service adjustments that may be required (Daatland and Herlofson 2003).

Despite several important cross-national studies (Daatland and Herlofson 2003; Daatland et al. 2011; Yeh et al. 2013), we still do not have detailed evidence on elder support “norms” (at the world region and country levels) and “attitudes” (at the individual level) for a large number of countries. We use 2012 data from the International Social Survey Program (ISSP) to examine variation in cultural norms and attitudes regarding assistance with daily activities for older people across economically developed countries (*N *= 25 countries for the descriptive analysis, *N *= 21 countries for the multilevel multinomial logistic regression analysis). We focus on cultural norms and attitudes regarding the appropriate roles of family members and formal providers in both the provision and financing of assistance with daily activities at home.

## Empirical evidence and hypotheses

Despite concerns about weakening elder support norms as societies “westernize” (Aboderin 2004), a comparison of findings from studies of economically developed countries suggests the existence of at least some cross-national differences in cultural norms regarding elder support (Cong and Silverstein 2012; de Valk and Schans 2008; Ganong and Coleman 2005; Killian and Ganong 2002; Takagi and Silverstein 2006). For example, co-residence norms appear to be significantly stronger in the USA (Burr and Mutchler 1999) than the Netherlands (de Valk and Schans 2008). De Valk and Schans (2008) report that only 3 percent of native Dutch respondents agreed with the statement that “if parents are old, children should provide co-residence for them.” Nevertheless, comparisons are difficult because of methodological differences between studies.

Several studies have examined differences in elder support norms across a small number of countries or societies using comparable measures and samples (Daatland and Herlofson 2003; Daatland et al. 2011; Daatland and Lowenstein 2005; Lowenstein and Daatland 2006; Yeh et al. 2013). Most of these studies use data from the OASIS project (Daatland and Herlofson 2003; Daatland and Lowenstein 2005; Lowenstein and Daatland 2006), a study of older people’s quality of life among urban populations in four European countries and Israel (Lowenstein and Ogg 2003). Daatland and Herlofson (2003) examine three dimensions of elder support norms: “filial obligation norms,” “preferences for care,” and “welfare state orientations.” The measures used in this study are most similar to Daatland and Herlofson’s (2003) measures of welfare state orientations. Studies suggest that the pattern of cross-national variation depends on the dimension of elder support norms examined. While filial obligation norms are stronger toward the South East of Europe and weaker toward the North West of Europe (Daatland and Herlofson 2003; Daatland et al. 2011; Daatland and Lowenstein 2005), people’s care preferences and welfare state orientations are “more or less congruent with national family and social policy traditions” (Daatland and Herlofson 2003:537).

While the studies cited in the previous paragraph demonstrate the existence of cross-national differences in elder support norms, their conclusions regarding macrolevel factors are speculative because of insufficient degrees of freedom at the country level. We estimate multilevel multinomial logistic regression models (*N *= 21 countries) to examine the importance of two sets of country-level factors—“macrostructural factors” and “cultural–contextual factors”—in explaining individuals’ elder support attitudes. The inclusion of non-European OECD countries in our analysis also enables us to provide a more complete description of cross-national differences in cultural norms regarding elder support than previous studies.

### Macrostructural factors

Our basic hypothesis regarding the effects of macrostructural factors such as the social policy context and labor market composition on individuals’ elder support attitudes is that, consistent with research on intergenerational solidarities (Bengtson and Roberts 1991; Daatland and Lowenstein 2005), we expect to find some correspondence between attitudes and social structure. With regard to the social policy context, previous research suggests that public support for formal services constitutes an important macrostructural factor influencing the feasibility of different elder support arrangements. Studies that rely on broad measures of informal assistance and use an instrumental variable approach to account for the endogeneity of informal assistance suggest that the “crowding out” or substitution effect predominates in the relationship between informal care and formal services (Bolin et al. 2008; Bonsang 2009; Gannon and Davin 2010; Van Houtven and Norton 2004) despite significant evidence of mixed responsibility in some countries (Litwin and Attias-Donfut 2009; Motel-Klingebiel et al. 2005). Therefore, we expect that in countries with greater public social expenditures on formal services people are more likely to prefer *publicly* financed formal assistance (**H1**). We also expect that in countries with greater expenditures on cash transfers people are more likely to prefer *privately* financed formal assistance (**H2**), because cash transfers increase household income and therefore encourage the purchase of privately financed services (Geerlings et al. 2005; Janus and Ermisch 2015; Van Groenou et al. 2006).

With regard to labor market composition, we hypothesize that in countries with a greater supply of women in the labor market, people are less likely to prefer assistance provided by informal helpers (i.e., “privately financed informal assistance” and “publicly financed informal assistance”) (**H3**). There may be both supply side and demand side mechanisms through which women’s labor force participation and formal care provision are related. On the supply side, a rise in women’s labor force participation and a resultant increase in the number of care workers could make formal care more available and cheaper. On the demand side, consistent with the literature on employment and informal caregiving (Henz 2006), a rise in women’s employment could reduce their availability for caregiving activities in the family and therefore increase demand for formal care.

### Cultural–contextual factors

Previous studies suggest the importance of people’s values, ideals, and beliefs *at the individual level* in explaining elder support attitudes and behaviors (Gans et al. 2009; Killian and Ganong 2002; Myers 2004). However, despite cross-national differences in political, religious, and familial norms (Blekesaune and Quadagno 2003; Pascall and Lewis 2004; Reher 1998), few studies have explicitly examined the possible impact of such cultural differences in explaining individuals’ elder support attitudes and behaviors (Haberkern and Szydlik 2010; Suanet et al. 2012). Pfau-Effinger (2005) argues that culture and policy should be analyzed as separate and independent dimensions and that the inter-relationship between the two is complex in that it is mediated by the macro- or societal context. Because religious teachings (e.g., the Ten Commandments) and organizational aspects of religion encourage a sense of filial obligation (Gans et al. 2009; Myers 2004), we hypothesize that individuals living in more religious countries will be more likely to prefer assistance provided by informal helpers (i.e., “privately financed informal assistance” and “publicly financed informal assistance”) (**H4**). Furthermore, consistent with research on people’s political values and support for the welfare state (Jaeger 2008; Roosma et al. 2016), we hypothesize that individuals living in more politically conservative countries will be more likely to prefer privately financed informal assistance and privately financed formal assistance (**H5**).

## Methods

### Data

We use data from the ISSP’s 2012 Family and Changing Gender Roles IV module. The descriptive analysis of differences in cultural norms by world region and country is based on 29,355 persons residing in 25 OECD countries: five Nordic countries (i.e., Denmark, Finland, Iceland, Norway, and Sweden); five English-speaking countries (i.e., Australia, Canada, Ireland, the UK, and the USA); four Western European countries (i.e., Austria, France, Germany, and Switzerland); five countries in other parts of Europe (i.e., Czech Republic, Poland, Slovakia, Slovenia, and Spain); two East Asian countries (i.e., Japan and Korea), as well as Chile, Israel, Mexico, and Turkey. The multilevel multinomial logistic regression analysis in which we examine the effect of macrolevel factors on individuals’ attitudes is based on 24,133 persons residing in the 21 countries for which we have data on all of the independent variables used in the analysis.

While all countries used national probability sampling, there were some differences in survey methodology including the lower and upper age cutoffs used to define the population, whether complex sample designs were used (e.g., stratification and multistage sampling), and mode of administration (see Gendall et al. (2016) for a detailed comparison of methodological differences between surveys). To increase comparability with respect to age, we restricted the analysis to respondents aged 18–79. There are two countries that represent narrower age ranges—Finland (ages 18–74) and Switzerland (ages 19–79)—which should be kept in mind in interpreting the results for these countries. The average country response rate was 53 percent, with countries using face-to-face interviewing generally having higher response rates. We used the survey weight provided with the data set to adjust for unequal probabilities of selection due to nonresponse and the use of complex survey designs.

### Dependent variable: elder support attitudes

**Fig. 1:**
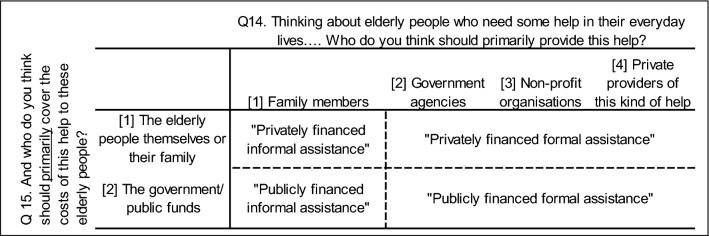
Coding scheme for dependent variable in multilevel regression analysis with question text and answering categories for questions on assistance

The dependent variable in the multilevel multinomial logistic regression analysis is based on respondents’ answers to two questions about who should provide and cover the costs of personal assistance with daily activities for older people. The question about who should provide help asks, “Thinking about elderly people who need some help in their everyday lives, such as help with grocery shopping, cleaning the house, doing the laundry. Who do you think should primarily provide this help?” Immediately following this question, respondents are asked, “And who do you think should primarily cover the costs of this help to these elderly people?” The dependent variable was coded as “publicly financed formal assistance,” “privately financed informal assistance,” “privately financed formal assistance,” or “publicly financed informal assistance” according to the coding scheme in Fig. 1, which also shows the verbatim answering categories for each question. Our measures of cultural norms regarding elder support, which were used in the descriptive analysis of differences by world region and country, were constructed based on the individual-level measure of respondents’ elder support attitudes and represent the percentage of respondents in a country (or world region) who are in favor of each of the four elder support arrangements.

### Country-level variables

#### Macrostructural factors

These country-level factors include indicators of the social policy context and labor market composition. Indicators of the social policy context include social expenditures by public institutions (at different levels of government) in two (of nine) policy areas in the OECD Social Expenditure Database (SOCX): old-age benefits and incapacity-related benefits (Adema et al. 2011). SOCX provides separate indicators for expenditures on services and cash transfers, which we used for our analysis. Examples of services include day care, rehabilitation, home help, and residential care services. Examples of cash transfer programs include old-age pensions and disability cash benefits paid to individuals who are unable to engage in paid work (Adema et al. 2011). Per head expenditures in US dollars were adjusted using purchasing power parities and expressed as the percentage of a country’s GDP. Our indicator of labor market composition is the percentage of the labor force that is female based on information from the OECD.Stat Web site (OECD 2018, August 14) (see Table 1 for countries’ values on country-level variables).

**Table 1:** Countries’ values on country-level variables (*n *= 24,133)

	Macrostructural factors			Cultural–contextual factors			Control variables	
	Services (% GDP)^a^	Cash transfers (% GDP)^a^	% Labor force female^a^	% With religious affiliation^a^	Religious attendance (%)^b^	% With leftist orientation^c^	Long-term care (% GDP)^a^	Dependency ratio^a^
Australia	2.1	5.4	45.6	66.9	Missing	49.6	0.1	20.6
Austria	1.0	12.9	46.7	86.5	22.3	38.8	1.2	26.1
Canada	0.0	4.8	47.4	48.9	22.5	57.1	1.2	20.9
Czech Republic	0.3	9.5	43.6	29.2	9.4	55.1	0.3	22.9
Denmark	3.5	9.2	47.4	83.8	5.9	49.1	2.3	26.2
Finland	2.2	11.7	47.6	78.5	9.6	46.7	0.6	27.1
France	0.4	13.4	47.7	55.6	10.1	57.4	1.2	26.5
Germany	0.7	9.6	46.2	65.5	16.1	61.5	0.9	31.7
Iceland	1.2	4.0	47.6	85.0	5.9	62.5	1.6	18.7
Ireland	0.7	6.0	44.6	88.7	49.1	72.8	0.4	17.3
Japan	2.0	9.6	42.1	35.2	8.2	67.3	0.8	36.6
Mexico	0.0	1.6	37.5	94.3	70.5	71.7	0.0	9.8
Norway	2.5	8.4	47.3	80.7	7.7	59.1	2.3	23.1
Poland	0.2	11.0	44.8	90.0	69.1	52.5	0.4	19.1
Slovak Republic	0.5	7.8	44.2	87.4	50.6	72.8	0.0	17.6
Slovenia	0.4	11.6	45.9	70.5	21.9	55.6	0.8	24.0
Spain	1.0	10.4	45.1	77.6	22.6	55.4	0.6	25.4
Sweden	4.3	8.7	47.3	76.9	7.9	61.8	0.6	28.8
Switzerland	0.9	7.7	45.7	75.0	19.0	32.3	1.4	25.1
UK	1.0	7.2	46.2	51.4	15.6	46.0	0.9	25.1
USA	0.0	7.4	46.6	78.5	44.7	58.7	0.6	19.8

^a^See independent variables section of the methods for more information on how this measure was constructed

^b^Percentage of the population attending religious services at least once per month

^c^Percentage of the population that is “far left,” “left” or “center” in political orientation

#### Cultural–contextual factors

These country-level factors are constructed based on the individual-level ideological factors. We tried two different indicators of the religious context in the analysis and show results for both. The first is based on religious affiliation and is the percentage of the population with any religious affiliation (e.g., “Catholic”). The second indicator is based on religious attendance and is the percentage of the population attending religious services at least once a month. Our indicator of the political context is based on the political orientation variable and is the percentage of the population who is far left, left or to the center in political orientation (Table 1).

#### Country-level control variables

We also control for countries’ public long-term health care expenditures and the old-age dependency ratio at the country level. The indicator of countries’ public long-term health care expenditures is based on information from the OECD.Stat Web site (OECD 2018, August 14) and, like the social expenditure indicators, is expressed as per head expenditures in US dollars as the percentage of a country’s GDP and is adjusted using purchasing power parities. The old-age dependency ratio is the percentage of those 65 and over divided by those who are between 15 and 64 years old and is based on information from the OECD.Stat Web site (OECD 2018, August 14) (Table 1).

### Individual-level control variables

We control for several ideological and socio-demographic factors at the individual level. Previous research suggests that such factors are related to individuals’ elder support attitudes (Killian and Ganong 2002), and, as a result, failure to control for these factors could bias our estimates of the effects of macrostructural and cultural–contextual factors.

The variables we control for include age; gender; a 5-category self-reported health status variable (“excellent,” “very good,” “good,” “fair,” “poor”); a 3-category educational attainment variable (“less than secondary,” “secondary,” “tertiary”); a binary marital status variable; a binary parental status variable; a 5-category urbanicity variable (“large city,” “suburbs,” “town,” “village,” “country”); a binary variable indicating whether a respondent reports any religious affiliation; a 4-category religious attendance variable (“at least once a week,” “at least once a month,” “less than once a month,” “never”); and a 7-category political orientation variable (“far left,” “left,” “center,” “right,” “far right,” “other affiliation,” “did not vote”). The three-category educational attainment variable is a recode of a more detailed variable constructed by ISSP to facilitate international comparison. To construct the parental status indicator, we used a question on respondents’ employment status when a child was under school age as well as two questions on household composition, because this module of the ISSP does not have a question that directly asks about the respondent’s parental status or number of children (details available from the first author upon request). Political orientation measures the party the respondent voted for in the last general election and was placed on a left–right scale by ISSP to facilitate international comparison (see Online Resource 1 for descriptive statistics of the individual-level variables).

### Missing data

We excluded respondents from Chile, Israel, Korea, and Turkey from the multilevel multinomial logistic regression analysis (i.e., applied casewise/listwise deletion), because the question on which the political orientation variable was based was not asked in these countries. We excluded these respondents rather than imputing political orientation, because accurately imputing this variable would be difficult in the absence of cases from the same country (and context) with nonmissing values on this variable. Of the 24,133 respondents from the remaining 21 countries, 4166 respondents (17 percent) had missing values on at least one of the individual-level variables (and in this case there were other respondents from the same country with nonmissing values that could be used in the imputations). We imputed missing values using multiple imputations (10 sets of imputations) with chained equations (White et al. 2011). In the imputation models, we included the full set of individual-level variables as well as indicator variables for each country (minus an indicator variable for the reference country) to take into account (or control for) the country context.

### Analytical approach

We use multilevel multinomial logistic regression analysis to examine the relationship of two sets of country-level factors—“macrostructural factors” and “cultural–contextual factors”—with individuals’ elder support attitudes. Therefore, countries are the level-2 units, while individuals are the level-1 units. “Publicly financed formal assistance” is the reference category. Our models control for ideological and socio-demographic factors at the individual level. The variables for age and gender have both fixed effects (as expressed by the regression coefficients or odds ratios) and random effects, while the remaining individual-level variables have fixed effects only. We estimate two different models with alternative indicators of the religious context—(a) the percentage of the population with any religious affiliation (e.g., “Catholic”) as well as (b) the percentage of the population attending religious services at least once a month. Our results for the other individual-level and country-level factors (our main results) are based on the model with the religious affiliation measure. Respondents from Australia are excluded from the model with the religious attendance measure, because the question about religious attendance was not asked in this country.

## Results

### Variation by world region and country in elder support norms

**Fig. 2:**
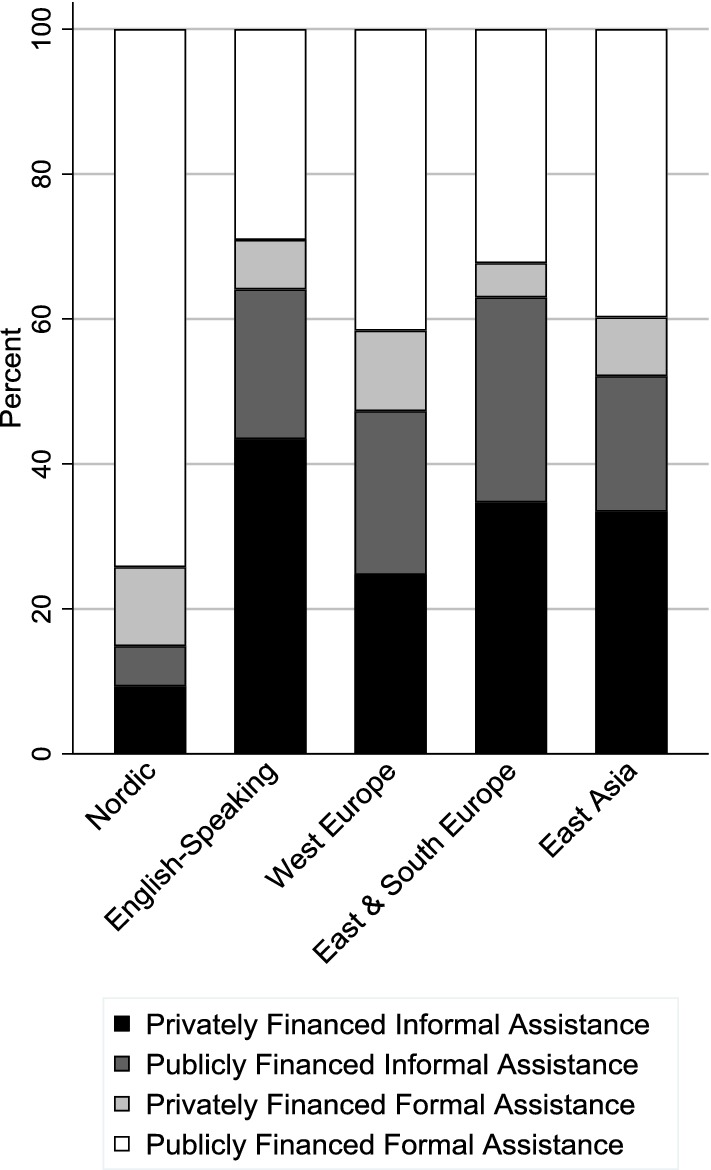
Support for different assistance arrangements by region

**Fig. 3:**
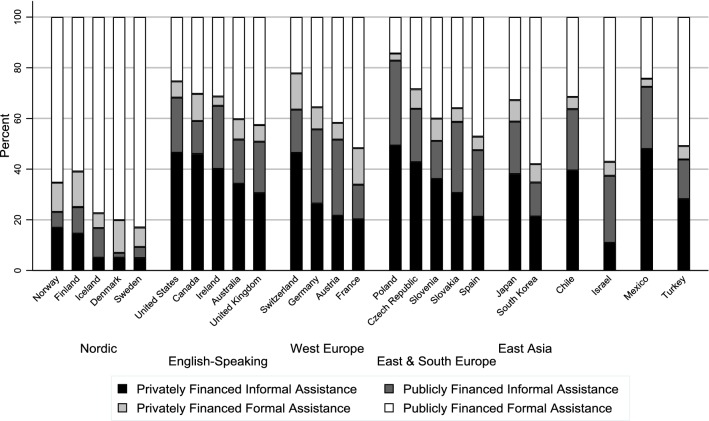
Support for different assistance arrangements by country

Among the 25 countries covered by the descriptive analysis of differences in cultural norms by world region and country, there is substantial variation in elder support norms both between regions (Fig. 2) and between countries (Fig. 3) within all regions except for the Nordic countries (see Online Resource 2 for percentage values by region and country). There is a clear divide between the Nordic countries and the rest of the world in terms of support for informal assistance arrangements (i.e., privately financed informal assistance and publicly financed informal assistance) (Fig. 2).

With regard to variation in elder support norms within regions (Fig. 3), among the English-speaking countries, informal assistance arrangements (i.e., privately financed informal assistance and publicly financed informal assistance) receive substantially greater combined support in the USA (68 percent), Ireland (65 percent), and Canada (59 percent) than Australia (52 percent) and the UK (51 percent). In Western Europe, combined support for informal assistance arrangements is greater than 50 percent in Switzerland (64 percent), Germany (56 percent), and Austria (52 percent). Support for publicly financed formal assistance is greatest in France (52 percent) among Western European countries.

Among Eastern and Southern European countries, support for privately financed informal assistance is greatest in Poland (49 percent) and the Czech Republic (43 percent), and support for publicly financed formal assistance is greatest in Spain (47 percent). Among the East Asian countries, support for publicly financed formal assistance is substantially greater in South Korea (58 percent) compared to Japan (33 percent).

Chile and Mexico appear similar to more “familialistic” countries such as Japan, Poland, and the USA in terms of combined support for informal assistance arrangements, while Israel and Turkey appear similar to less “familialistic” countries such as France, although support for publicly financed formal assistance is still less in Israel and Turkey than in any of the Nordic countries.

### Country-level factors

**Fig. 4:**
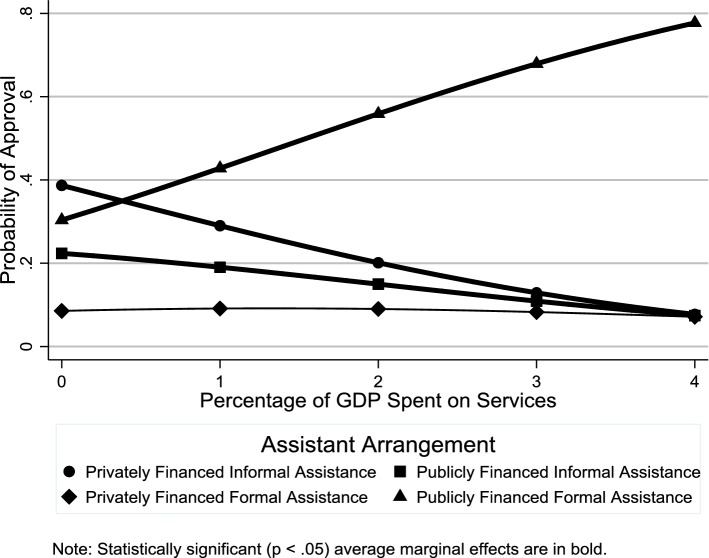
Average marginal effects of public expenditures on services on probability of support for difference assistance arrangements

**Fig. 5:**
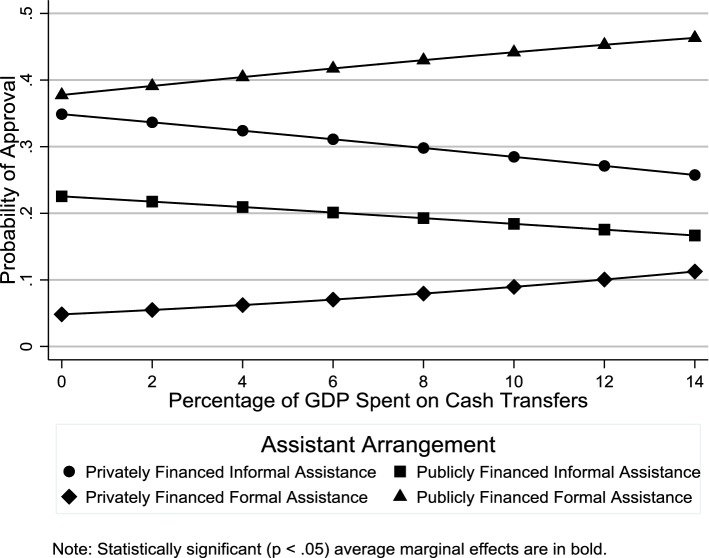
Average marginal effects of public expenditures on cash transfers on probability of support for different assistance arrangements

**Fig. 6:**
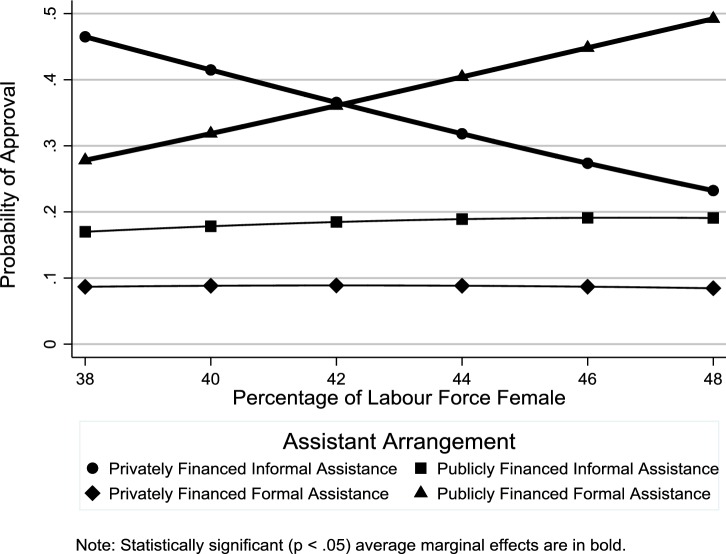
Average marginal effects of percentage of labor force that is female on probability of support for different assistance arrangements

In this section, we discuss results from the multilevel multinomial logistic regression analysis regarding the relationship of country-level factors with individuals’ elder support attitudes (Online Resource 3 shows odds ratio estimates for the individual-level control variables). Figure 4 shows the average marginal effects of public expenditures on services on the probability of support for different assistance arrangements. Consistent with **H1**, there was greater support for publicly financed formal assistance in countries with higher spending on services (see also Table 2). Contrary to **H2**, people living in countries with higher spending on cash transfers were not more likely to support privately financed formal assistance (Fig. 5 and Table 2: OR = 1.03, *p *≥ .05). Consistent with **H3**, there was less support for privately financed informal assistance in countries in which a greater percentage of the labor force was female (Fig. 6 and Table 2: OR 0.89, *p *< .05). While this result supports **H3**, the gender composition of the labor force had no such effect on support for publicly financed informal assistance (Fig. 6 and Table 2: OR 0.97, *p *≥ .05).

**Table 2:** Odds ratio estimates for country-level factors (*n *= 24,133)

	Privately financed informal assistance		Privately financed formal assistance		Publicly financed informal assistance	
	Odds ratio	(95% CI)	Odds ratio	(95% CI)	Odds ratio	(95% CI)
*Macrostructural factors*						
Services	0.51**	(0.41–0.64)	0.83*	(0.69–1.00)	0.53**	(0.45–0.64)
Cash transfers	0.92	(0.84–1.01)	1.03	(0.95–1.13)	0.90*	(0.83–0.97)
% Labor force female	0.89*	(0.79–0.99)	0.94	(0.85–1.04)	0.97	(0.88–1.07)
*Cultural–contextual factors*						
% With religious affiliation	1.00	(0.99–1.02)	0.99	(0.98–1.01)	1.02**	(1.01–1.03)
% Attending religious services at least once a month	1.02*	(1.00–1.04)	0.99	(0.98–1.01)	1.03**	(1.01–1.04)
% With leftist political orientation	0.99	(0.97–1.01)	0.99	(0.97–1.01)	0.99	(0.97–1.01)
*Country*-*level control variables*						
Long-term care	0.96	(0.61–1.52)	0.85	(0.57–1.28)	0.96	(0.66–1.40)
Dependency ratio	1.08*	(1.02–1.15)	1.01	(0.95–1.06)	1.14**	(1.08–1.20)

Estimates from multilevel multinomial logistic regression model with random intercepts and random effects for age and gender. The reference category is “publicly financed formal assistance”

*p < 0.05, **p < 0.01

**Fig. 7:**
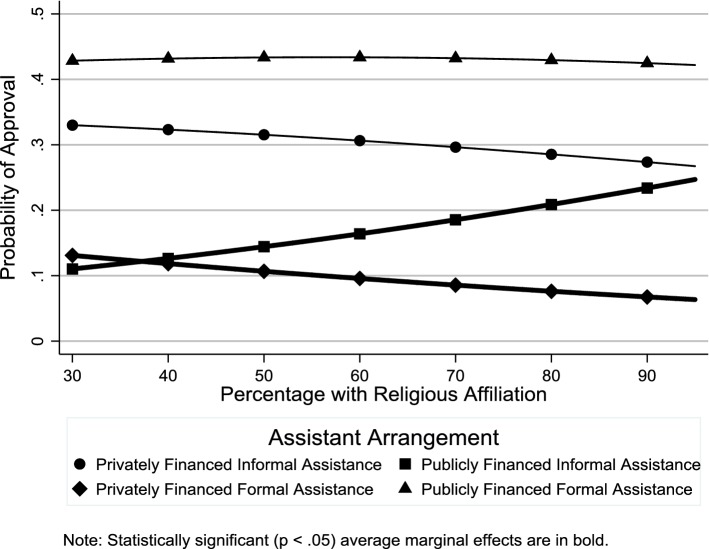
Average marginal effects of percentage with religious affiliation on probability of support for different assistance arrangements

**Fig. 8:**
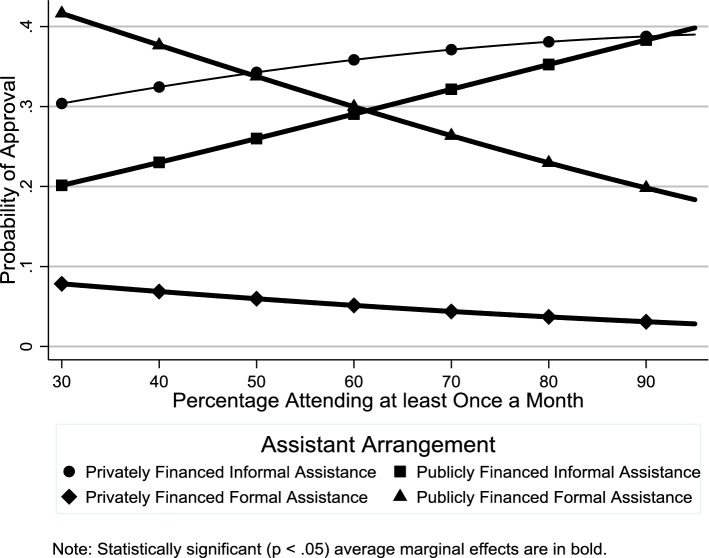
Average marginal effects of religious service attendance on probability of support for different assistance arrangements

**Fig. 9:**
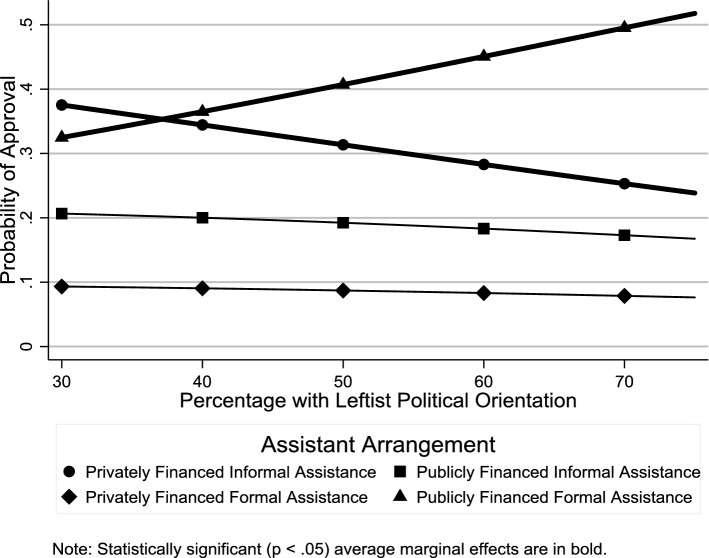
Average marginal effects of percentage with leftist political orientation on probability of support for different assistance arrangements

We tried two different indicators of the religious context in our analysis: percentage with a religious affiliation and percentage attending religious services at least once a month. With regard to the first indicator, consistent with **H4**, there was greater support for publicly financed informal assistance in more religious contexts (Fig. 7 and Table 2: OR 1.02, *p *< .01), although there was no such effect on support for privately financed informal assistance (Fig. 7 and Table 2: OR 1.00, *p *≥ .05). With regard to the second indicator, consistent with **H5**, there was greater support for publicly financed informal assistance in countries with higher levels of religious service attendance (Fig. 8 and Table 2: OR 1.03, *p* < .01). However, while both the odds ratio and average marginal effects indicate a positive relationship between religious service attendance and support for privately financed informal assistance, only the odds ratio was statistically significant (Fig. 8 and Table 2: OR 1.02, *p *< .05). According to the average marginal effects estimates in Fig. 9, consistent with **H6**, there was greater support for publicly financed formal assistance in countries with a left-leaning political context, although, contrary to **H6**, there was no such effect on support for publicly financed informal assistance.

### Estimates of variance components

Our multilevel multinomial logistic regression model has random intercepts and random effects for age and gender. The interclass correlation coefficients for each binary contrast range from 0.06 to 0.30 and can be interpreted as the proportion of variance that is accounted for by the country level (see estimates from the empty model in Online Resource 4). While the variance components for the country intercepts from the full model were substantially smaller in magnitude compared to the model with level-1 factors only, they remained statistically significant, which suggests that important level-2 factors remain unaccounted for in explaining individuals’ elder support attitudes (see Online Resource 4).

## Discussion

In summary, we find substantial variation in cultural norms both between world regions and between countries within all world regions except for the Nordic countries. The multilevel regression analysis points to the importance of two sets of country-level factors—“macrostructural factors” and “cultural–contextual factors”—in explaining individuals’ elder support attitudes.

Respondents’ perceptions of the broad categories of provision and financing arrangements that underlies their answers to the attitudinal questions were likely influenced by differences between countries in the elder support context, such as the tasks of family helpers (Brandt et al. 2009) and the direct and opportunity costs that family helpers are likely to face (Isengard and Szydlik 2012; Van Der Lippe et al. 2011). A possible future direction for cross-national research on attitudes and cultural norms regarding elder support would be, similar to some studies of single countries, the use of attitudinal items or vignettes that manipulate the context of elder support. Keeping limitations with our measures of attitudes and cultural norms in mind, we have the following conclusions.

First of all, with regard to the cross-national differences, our results confirm previous studies’ findings of substantial variation across countries in cultural norms regarding elder support (Daatland and Herlofson 2003; Daatland et al. 2011; Daatland and Lowenstein 2005), with one exception: low levels of support (no more than 15 percent of respondents) for privately financed formal assistance across all of the countries covered by the analysis. Furthermore, the pattern of cross-national variation is complex in that it does not easily conform with existing welfare state typologies (Bettio and Plantenga 2004; Jensen 2008) or the Northwest/Southeast division that Daatland et al. (2011) use to characterize variation in filial and parental responsibility norms, although we do find that the Nordic countries are distinguished by very high levels of support for publicly financed formal assistance.

In an effort to make sense of substantial, complex variation in cultural norms regarding elder support, in the multilevel regression analysis, we examine the influence of two sets of country-level factors—“macrostructural factors” and “cultural–contextual factors”—on individuals’ elder support attitudes. We find that aspects of both are important. Consistent with our hypotheses, we find greater support for publicly financed formal assistance in countries with higher spending on services. The fact that people are less likely to prefer informal assistance arrangements in countries with greater service expenditures does not necessarily imply that family solidarity is weaker in such contexts. Individuals’ elder support attitudes are strongly dependent on the type of support considered, and people are generally more likely to prefer that socioemotional support is provided by family members compared to other forms of support, such as assistance with daily activities, across contexts (de Valk and Schans 2008; Lin and Yi 2011). Indeed, analogous to Brandt et al.’s (2009) argument regarding the effect of the availability of social and health services on intergenerational “help” and “care,” greater public provision may facilitate a “crowding in” of forms of support predominantly provided by family members such as socioemotional help.

We included public social expenditures on cash transfers as a factor in the analysis to capture the extent of countries’ reliance on market mechanisms in the delivery of elder support services (Brennan et al. 2012; Kvist 2012). Contrary to our hypotheses, we did not find that support for privately financed formal assistance was greater in countries with such market mechanisms. The absence of an “effect” for expenditures on cash transfers (as well as low support for privately financed formal services across countries) could be explained by the fact that in many cases cash allowances and tax concessions are insufficient to meet the costs of services and their generosity may depend on the economic resources of the recipient (Colombo et al. 2011). Finally, with regard to macrostructural factors, we found that in countries with a greater supply of women in the labor market there is less support for privately financed informal assistance (but not publicly financed informal assistance), which is partly consistent with our hypotheses.

The effects of the cultural–contextual factors are mostly consistent with our hypotheses and suggest the importance of taking into account the wider religious and political context in explaining individuals’ elder support attitudes. Pfau-Effinger (2005) notes that previous studies have included such cultural factors, either explicitly or implicitly, by examining how cross-national differences correspond to welfare state typologies, which partly reflect differences in the basic principles that shape social policy development. In our analysis, we take a “variable-centered” approach that involves modeling the effects of specific dimensions of the macrosocietal context on attitudes. Our results suggest that the influence of the cultural context is not restricted to its relationship via social policy development and that, as Pfau-Effinger (2005) suggests, constitutes an independent dimension that should be analyzed separately.

Finally, the statistically significant variance components for the country intercepts from the multilevel regression analysis suggest that our model omits important country-level factors. Due to data limitations and insufficient degrees of freedom at the country level, we were unable to include all possible country-level factors in our model. Possible omitted variables include family leave provision, migrant composition of the labor force, and support for gender egalitarian norms.

Overall, our results suggest that elder support norms are related, *yet irreducible*, to other dimensions of elder support such as macrostructural factors and the cultural context. For social scientists, this implies that they should take elder support attitudes (at the individual level) and cultural norms regarding elder support (at the macrolevel) into account to improve their explanations of differences in people’s actual elder support arrangements. Possible future research directions in this area include dealing with the endogeneity of elder support norms and attitudes (through, for example, the use of panel data that includes information on normative and attitudinal trends) and examining whether elder support norms and attitudes moderate the impact of macrostructural factors on elder support arrangements.

From a social policy perspective, because elder support norms constitute an independent dimension of elder support cross-nationally, this suggests that the elder support norms in a country should serve as a guideline for policy development. For example, the moderate to substantial support for arrangements in which assistance is provided by informal providers in all countries outside of the Nordic countries points to the usefulness of support allowances in potentially giving older people the flexibility to pay a formal or family provider. Substantial numbers of older people with support needs already receive such allowances in Germany, Poland, the UK, and several other countries (Lamura et al. 2008). However, informal providers’ access to support services such as counselling, respite care, and training is essential to reconciling some older people’s desire for family support with the well-being of informal helpers and policy imperatives to increase employment rates among women and older workers (Lamura et al. 2008).

## Electronic supplementary material

Below is the link to the electronic supplementary material.

Supplementary material 1 (DOCX 32 kb)
