# Supplementary material for: Whose responsibility? Elder support norms regarding the provision and financing of assistance with daily activities across economically developed countries
Source: Eur J Ageing. 2019 May 10;17(1):95–108. doi: 10.1007/s10433-019-00515-z (PMC7040143; doi:10.1007/s10433-019-00515-z)
Supplement: Supplementary file 1 — Supplementary material 1 (DOCX 32 kb) [file 10433_2019_515_MOESM1_ESM.docx]

# Electronic Supplementary Material

Article: Janus*, Alexander L., and Alison Koslowski. “Whose Responsibility? Elder Support Norms Regarding the Provision and Financing of Assistance with Daily Activities across 24 Countries.” *European Journal of Ageing* *University of Edinburgh, [alex.janus@ed.ac.uk](mailto:alex.janus@ed.ac.uk)

Online Resource 1. Descriptive Statistics for Individual-Level Variables (*n* = 24,133)

Online Resource 2. Percentage Supporting Different Delivery and Financing Arrangements by Region and Country (*n* = 29,355)

Online Resource 3. Odds Ratio Estimates^a^ for Individual-Level Control Variables (*n* = 24,133)

Online Resource 4. Estimates of Variance Components for Country Intercepts (*n* = 24,133)
